# Pancreatic infiltrative malignancy masquerading as autoimmune pancreatitis: Case report, review of radiological criteria, and literature

**DOI:** 10.1016/j.radcr.2024.05.025

**Published:** 2024-06-03

**Authors:** Hovhannes Vardevanyan, Martina Hager, Felix Renneberg, Rosemarie Forstner

**Affiliations:** a Department of Diagnostic Imaging, Armenian-American Wellness Center, Yerevan, Armenia; b Department of Pathology, University Hospital of Salzburg, PMU, Salzburg, Austria; c Department of Internal Medicine III, University Hospital of Salzburg, PMU, Salzburg, Austria; d Department of Radiology, University Hospital of Salzburg, PMU, Salzburg, Austria

**Keywords:** Autoimmune pancreatitis, Pancreas, Cancer, Masquerading, CT, MRI

## Abstract

We report a case of a 44-year-old male patient, who presented to the University Hospital of Salzburg, Austria with abdominal pain, persistent jaundice, and lack of appetite. Radiological work-up (CT, MRI, PET/CT) indicated a suspicious mass of the uncinate process of the pancreatic head with adjacent infiltration and regional lymphadenopathy. The differential diagnosis was between primary pancreatic cancer and focal autoimmune pancreatitis. Further laparoscopic biopsies from multiple areas, showed only fibrous scarring processes, with no malignancy. Treatment with steroids didn't give any benefits. After multiple follow-up CTs and MRs within 6 months—additional biopsies were done, which eventually demonstrated adenocarcinoma. Evidently the cancer diagnosis was much delayed and the patient started receiving chemotherapy, but radical surgery was not possible. Multiple articles and case reports can be found in the literature, that are reviewing the fact that pancreatic inflammatory processes are mimicking pancreatic tumor, but not many articles or case reports are available in the literature, where neoplastic processes are misinterpreted as inflammatory and incorrectly proven with histological examination. One of the main reasons for improper diagnosis is the desmoplastic reaction around the pancreatic malignancy. Another important aspect is the acceptance of histological diagnosis as conclusive, where no opposing arguments are specified, based on radiological criteria.

## Introduction

Pancreatic ductal adenocarcinoma is the most common malignant tumor of the pancreas and accounts for more than 85% of all pancreatic malignancies [8,9]. Pancreatic adenocarcinoma is highly resistant to current therapies, and 5-year overall survival rate is less than 7% [7,10]. This makes pancreatic adenocarcinoma the deadliest of all adult abdominal tumors [11,12]. Abdominal pain is the most frequently reported clinical symptom, even when the tumor is small (<2 cm), other symptoms include weight loss, pruritus, and jaundice [13]. Pancreatic cancer typically arises in the context of inflammation, and a surrounding area of pancreatitis is often present within the tumor microenvironment [14]. Radiological findings typical for pancreatic cancer are a hypovascular mass which causes pancreatic duct and common bile duct dilatation [4]. Many authors review pancreatic focal inflammatory changes that mimic pancreatic cancer, particularly IgG4-mediated autoimmune pancreatitis [1]. Our report represents a case of pancreatic cancer that was misdiagnosed as inflammatory with both histological and radiological examinations.

Autoimmune pancreatitis is a rare entity of unknown etiology that can mimic pancreatic cancer and whose diagnosis involve clinical, serological, radiological and histological findings [19]. There are 2 types of autoimmune pancreatitis: type 1, in which the pancreas is involved as one part of a systemic IgG4-related disease, and type 2, generally without IgG4-positive cells and without systemic involvement [20]. The classic clinical presentation in type 1 is painless obstructive jaundice (up to 75% of cases) mimicking pancreatic cancer. Other clinical features include chronic or recurrent abdominal pain (68%), acute pancreatitis, and steatorrhea [20]. On the other hand, type 2 autoimmune pancreatitis affects mainly younger patients, without a gender predilection, and often is not associated with hyper-IgG4 [21].

Peripancreatic vessel involvement is one of the characteristic biological behaviors of autoimmune pancreatitis, particularly for type 1 autoimmune pancreatitis, which hinders the diagnosis in differentiating autoimmune pancreatitis from pancreatic malignancies [6]. Takahashi et al. [5] showed that vascular involvement in patients with autoimmune pancreatitis was relatively common on CT, with a frequency similar to that of carcinoma.

In addition to extrapancreatic features (IgG4-related associated systemic disease: retroperitoneal fibrosis, periaortitis, inflammatory renal pseudotumors, autoimmune cholangiopathy and more), the lack of pancreatic duct dilatation is a key feature that distinguishes autoimmune pancreatitis from adenocarcinoma [15,16].

Several lines of evidence suggest that misdiagnosis, either radiologically or pathologically, may be relatively common [16]. Desmoplastic reaction adjacent to pancreatic cancer can be quite severe which can be misleading on pathological examinations [2,3]. Published false-negative rates for the pathologic misdiagnoses of pancreatic adenocarcinoma range from 1.6% to 30% [17]. In a study of 25 patients who survived more than 5 years after surgical resection for adenocarcinoma, 13 failed to show the typical pathologic characteristics of adenocarcinoma on retrospective analysis [18]. In a series of 186 patients diagnosed with adenocarcinoma who underwent resection, 12 had their diagnosis changed on further pathologic review [18].

### Case report

We report a 44-year old patient's case, admitted with abdominal pain, jaundice, and lack of appetite. A month before the current clinical work-up, the patient was diagnosed with drug-induced hepatitis (citerizine). Laboratory data showed high levels of: Bilirubin (direct) – 0.8 mg/dL, Gamma-GT - 1896U/l, ALT - 622U/l, AST - 239U/l, Alk. Phosphatase – 270 U/l. Due to persistent cholestasis—abdominal CT was indicated.

**Fig. 1:**
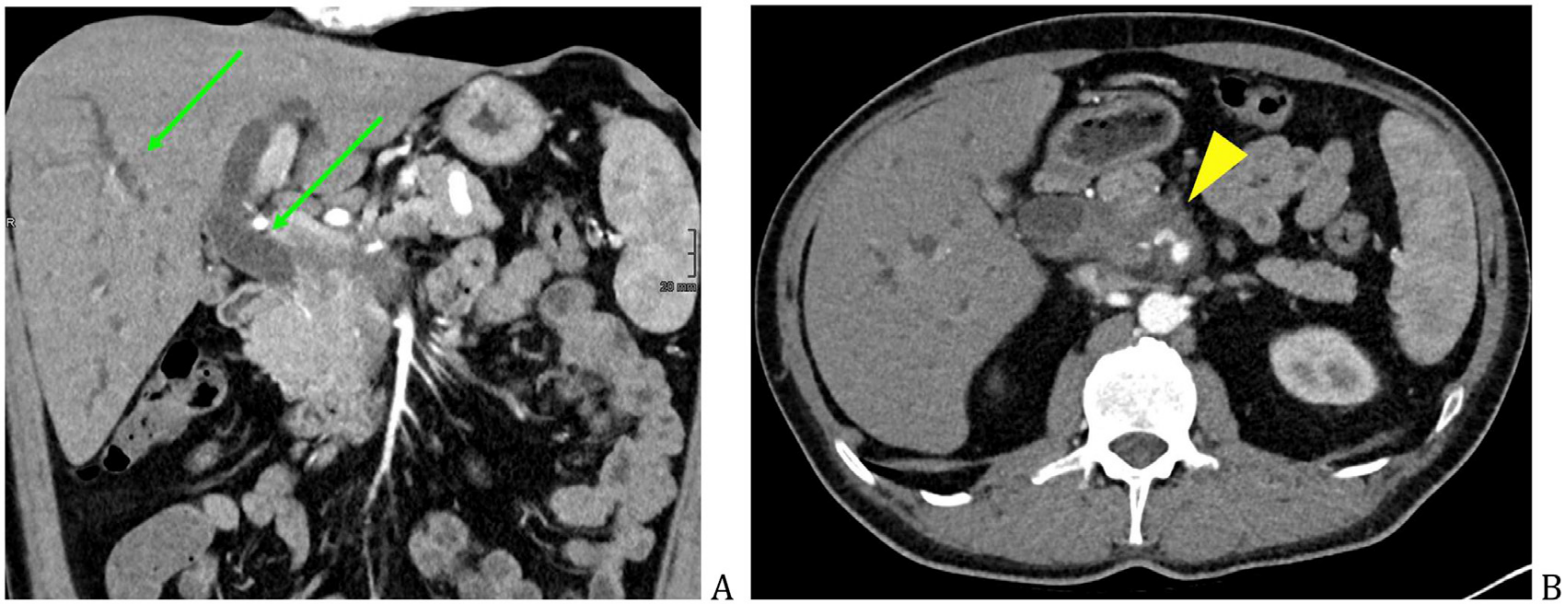
Initial CT, intra- and extrahepatic biliary dilatation (green arrow) (A), caused by a hypovascular mass around the superior mesenteric artery (yellow arrowhead) (B).

On CT, marked intra- and extrahepatic biliary dilatation, with severe stenosis of the distal common bile duct was noted (Fig. 1A), a suspicious soft-tissue mass was described around the pancreatic head, with circumferential encasement of superior mesenteric vessels, celiac trunk, and the portal vein (Fig. 1B). No pancreatic atrophy or Wirsung duct dilatation was seen (Figs. 7A, 8A, and B). Enlarged peripancreatic lymph nodes were present, mainly in the hepatoduodenal ligament, up to 13 mm in short axis.

**Fig. 2:**
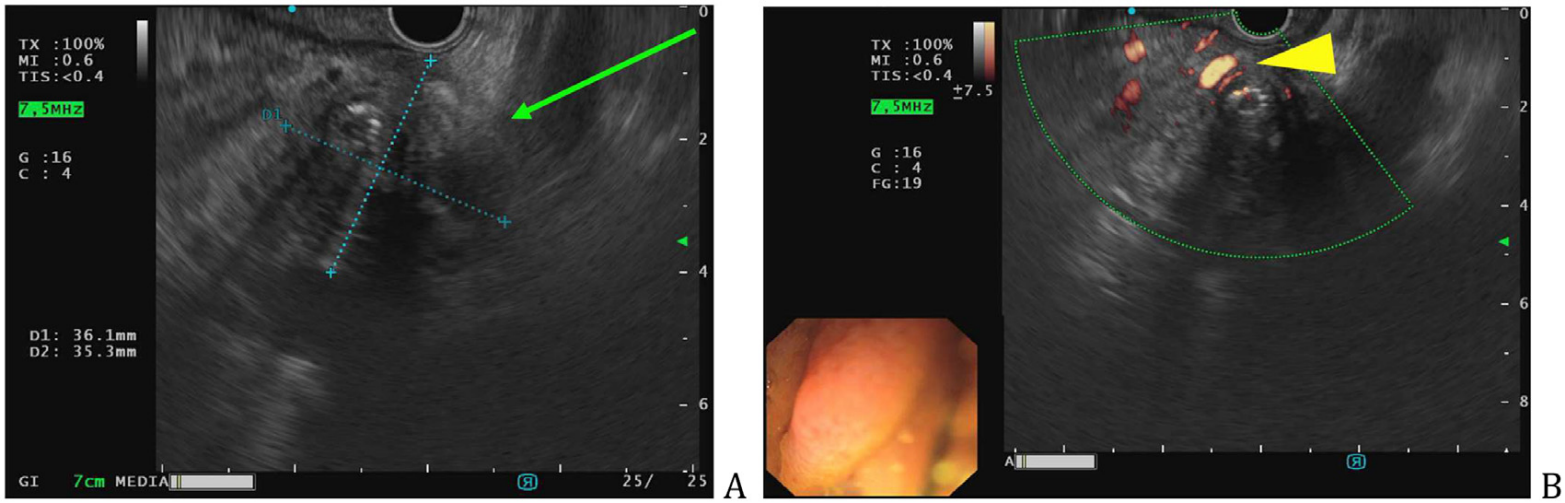
Endoscopic ultrasound showing pathological mass around the pancreatic head (green arrow) (A), with signs of peripheral blood flow (yellow arrowhead) (B).

Metal mesh stent was set in the common bile duct under ERCP. EUS of the pancreatic mass (Figs. 2A and B) with fine needle aspiration biopsy was performed and further cytological examination showed no evidence of malignancy.

**Fig. 3:**
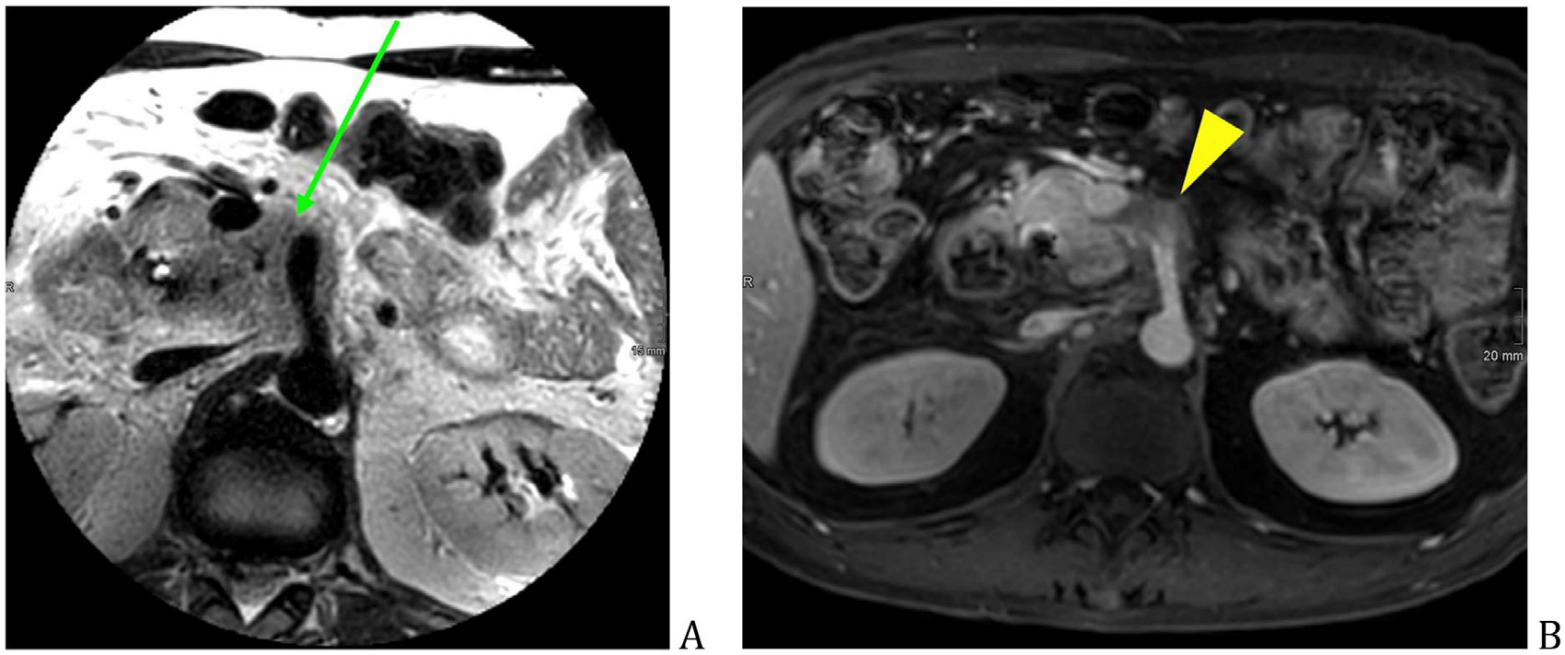
Pancreatic head mass, intermediate T2 signal on MR (green arrow) (A), poor enhancing soft-tissue mass on postcontrast T1 FS (yellow arrowhead) (B).

**Fig. 4:**
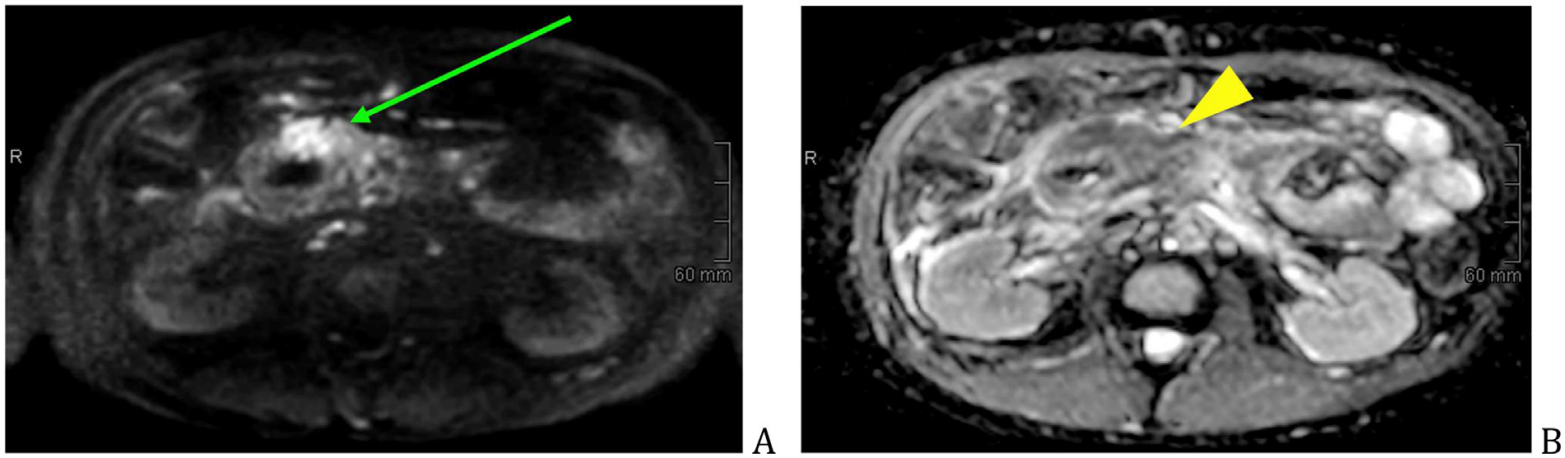
Pancreatic head mass restricted on DWI (green arrow) (A), with low-values of ADC (yellow arrowhead) (B).

Abdominal MRI and MRCP was also performed, which showed mild relief of the biliary dilatation (after stenting), minimal reactive fluid was seen in the right upper abdomen. The previously-described pancreatic head mass (Fig. 3A) was causing adjacent vessel encasement (Fig. 3B) and was restricted on DWI (Figs. 4A and B). Pancreatic cancer or Type 1 autoimmune pancreatitis was suspected, without liver metastasis.

Exploratory diagnostic laparoscopy was performed, multiple tissue specimens were taken from the pancreas head mass, the lesser omentum, and periportal abdominal lymph nodes, which showed no malignancy (Fig. 9), fibrosis and chronic inflammation was present histologically (Fig. 10).

**Fig. 5:**
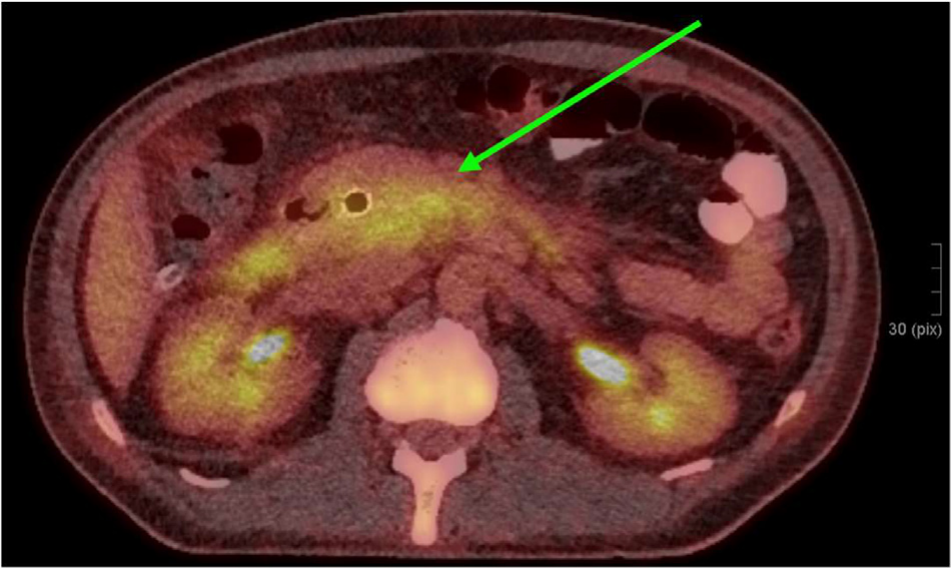
PET/CT, FDG-avid zone around the pancreatic head (green arrow).

PET/CT was performed to rule out dissemination. Only the pancreatic mass was uptaking FDG, which was to be differentiated between autoimmune pancreatitis or cancer (Fig. 5).

Corticosteroid treatment was performed for 3 weeks, which did not show any therapeutic effect.

IgG level was below normal-560 mL/dL, IL-6 was high–304.0 pg/mL. Serum amylase and lipase were later elevated – 721U/l and 1396 U/l respectively. Rituximab therapy was started, because type 1 autoimmune pancreatitis was considered diagnosed. The patient further complained of deterioration of his condition, which was because of pancreatitis.

**Fig. 6:**
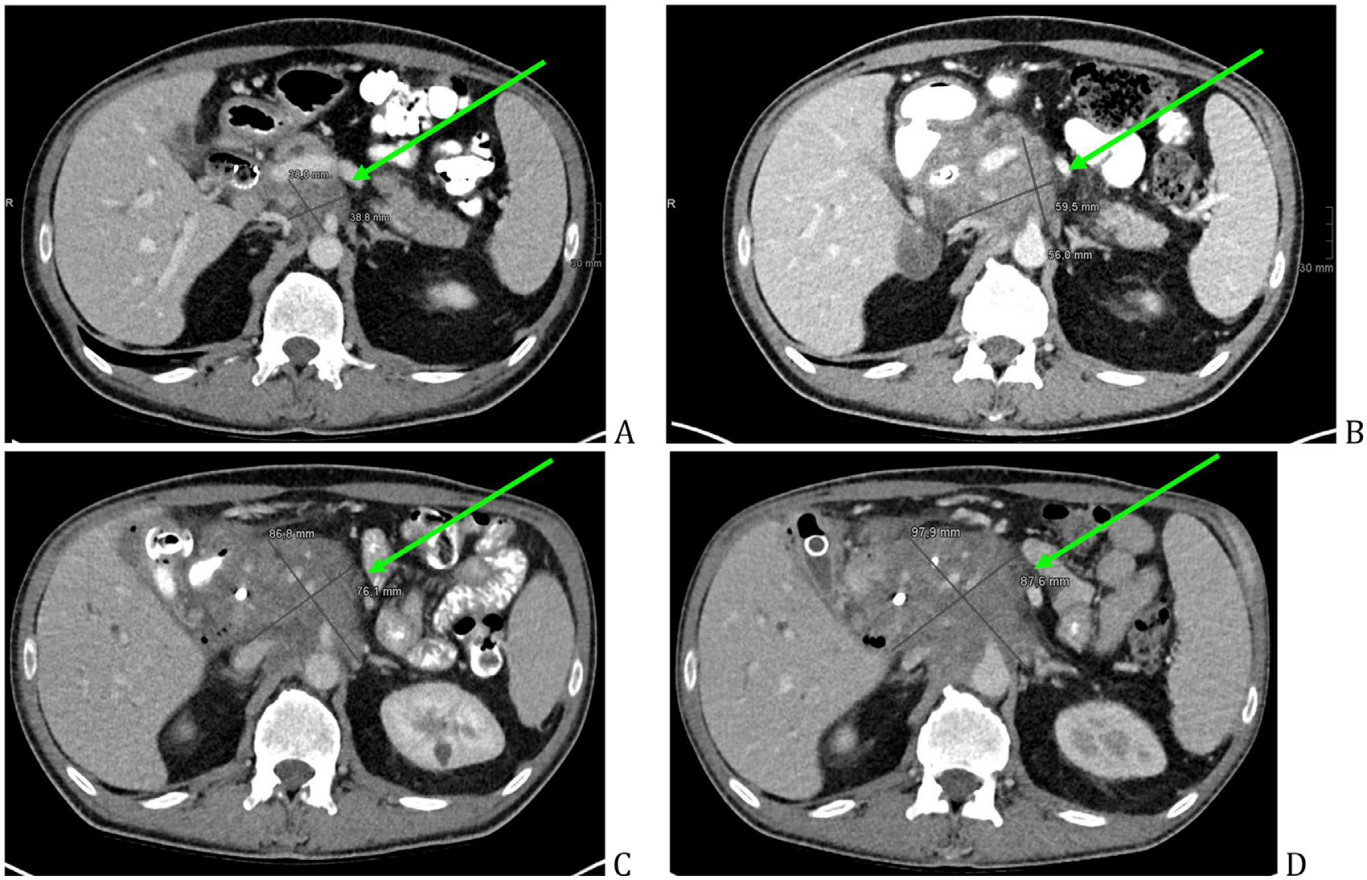
Follow-up CT within 6 months, pancreatic head mass (green arrow) size increase. Initial work-up - 38 mm (A), follow-up after 3 months—59 mm (B), follow-up after 5 months—86 mm (C), follow-up after 6 months—98 mm (D).

**Fig. 7:**
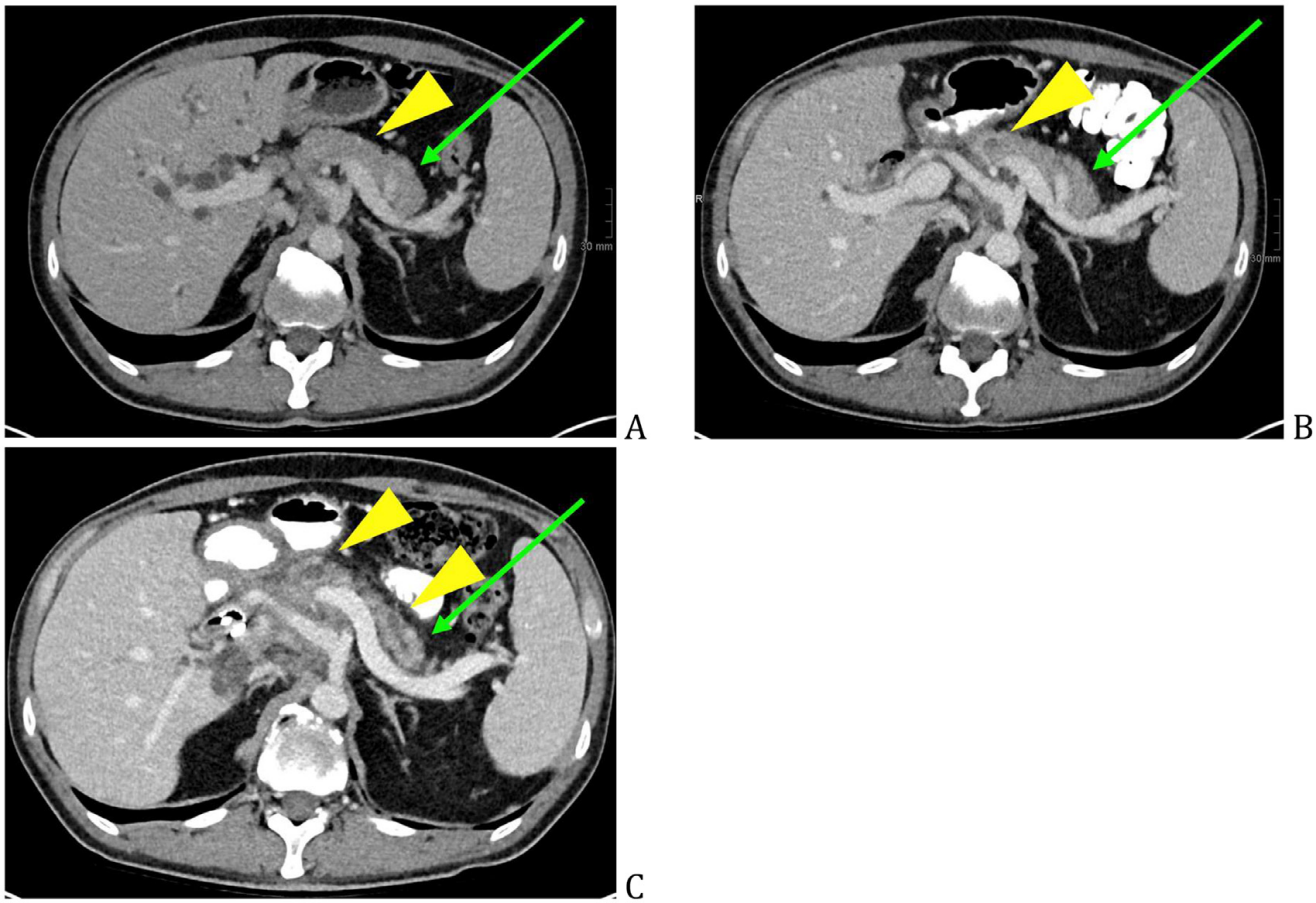
Follow-up CT - span of 4 months, pancreatic tail atrophy (green arrow) and main pancreatic duct upstream dilatation (yellow arrowhead). Initial work-up—no pancreatic atrophy and no duct dilatation (A), follow-up after 1 month—early signs of pancreatic tail atrophy and main pancreatic duct minimal dilatation (B), follow-up after 4 months—evident pancreatic tail atrophy and marked main pancreatic duct dilatation (C).

**Fig. 8:**
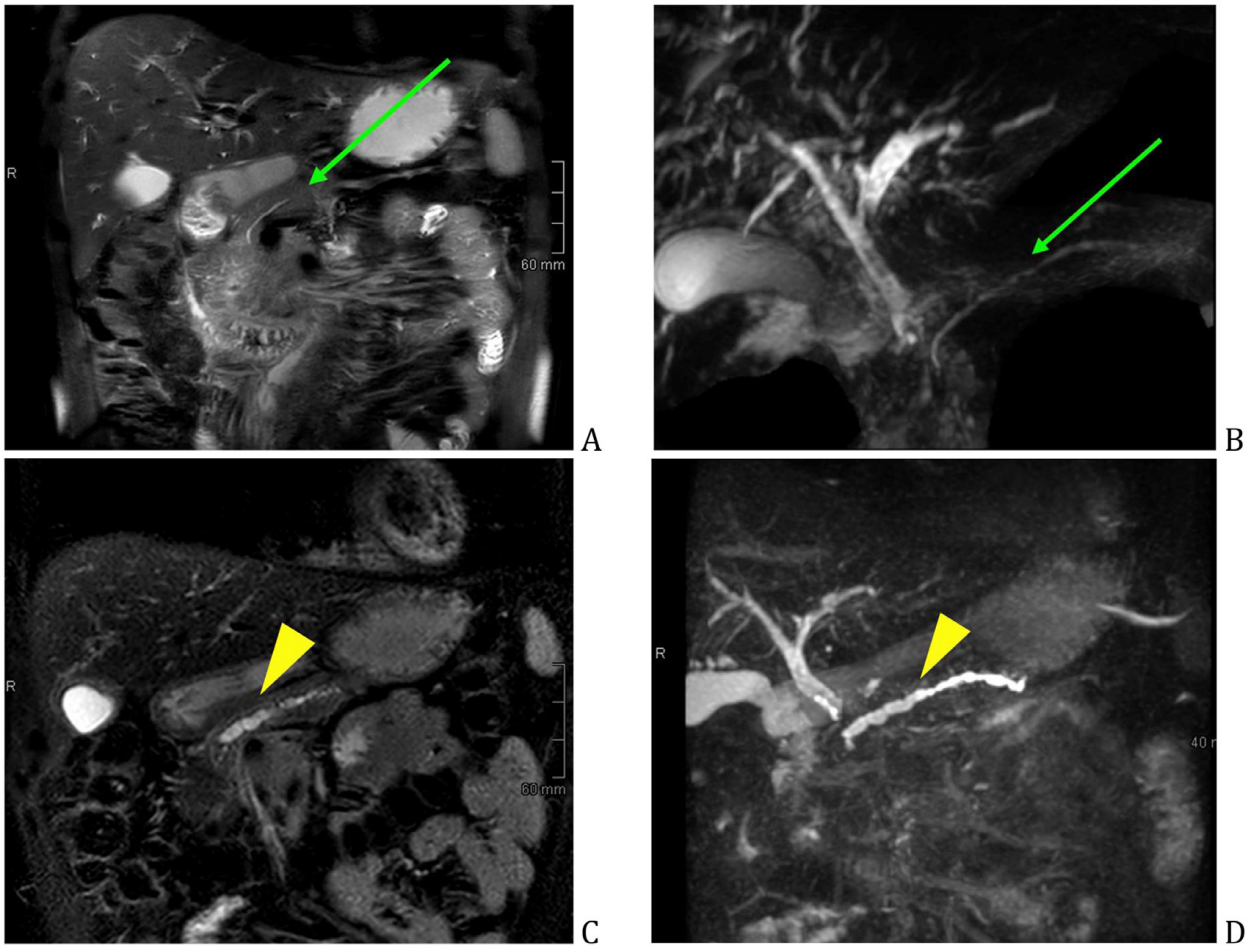
Follow-up MRI/MRCP - span of 2 months, main pancreatic duct dilatation. Initial work-up – no main pancreatic duct dilatation (green arrow) (A, B), follow-up after 2 months—apparent main pancreatic duct dilatation (yellow arrowhead) (C, D).

Within a 6-month period, multiple cross-sectional imaging examinations (CT, PET/CT, MRI, MRCP) were performed, the size increase of the pancreatic mass with adjacent infiltration was evidently seen on the images (Figs. 6A-D), which lead to diagnostic reevaluation and rebiopsy. Other evident CT and MR criteria that are highly suspicious for pancreatic cancer were: main pancreatic duct upstream dilatation (Figs. 7A-C, 8C and D), pancreatic tail atrophy (Figs. 7A-C), soft-tissue extensive growth around the peripancreatic vessels, portal vein severe stenosis and upper abdominal lymphadenopathy. The cholestasis was minimally deteriorating, the mesh stent was removed, double-pigtail stent was put instead in CBD, and gastro-cholecystostomy was created by hot axios stent.

**Fig. 9:**
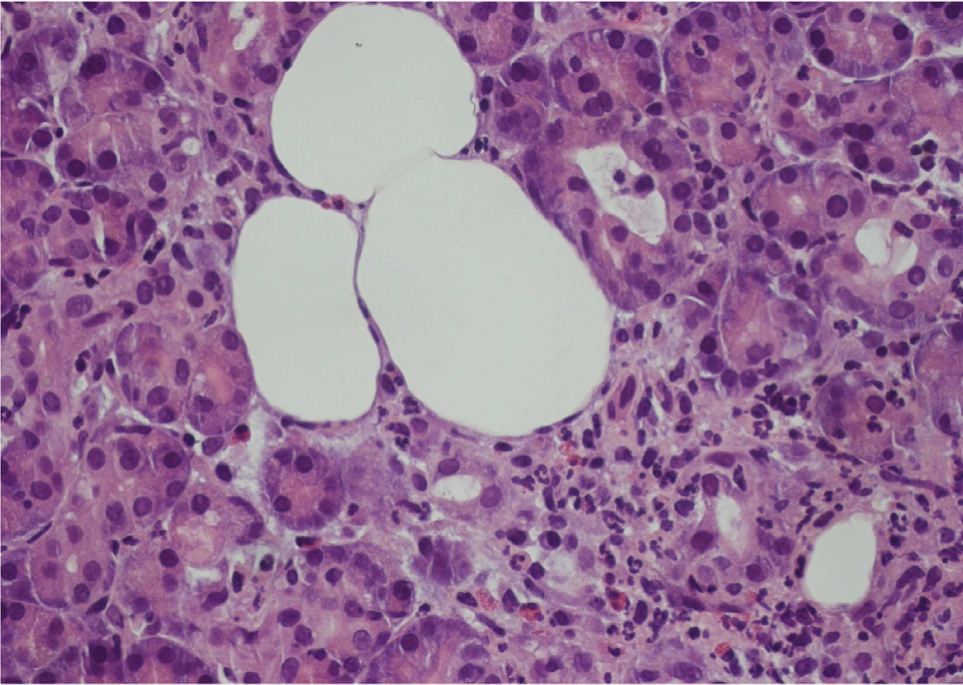
Acinar parenchyma of the pancreas, infiltration of neutrophils and some eosinophil granulocytes. Haematoxylin eosin stained, magnification 400x.

**Fig. 10:**
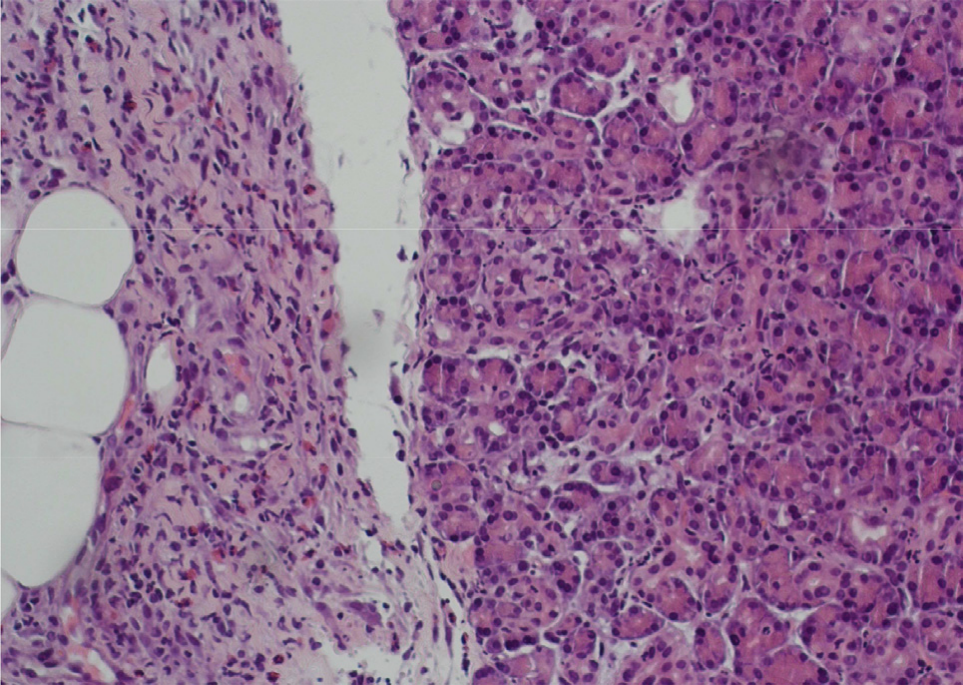
Acinar pancreatic parenchyma and adjacent fibrosis with infiltration of neutrophils and some eosinophil granulocytes. Hematoxylin eosin stained, magnification: 200x.

After 6 months from the diagnostic laparoscopy, the patient's condition was deteriorating and radiologically negative changes were evident, resembling pancreatic cancer. Biopsy was repeated from the pancreatic head mass, which showed adenocarcinoma. Treatment was continued with 5 cycles of FOLFIRINOX, showing some decrease in tumor size. Cholangitis and cholesepsis further developed with intrahepatic biliary abscess formation.

## Discussion

This case presents an interesting clinical constellation, when radiological examinations indicate a pancreatic head mass, with adjacent vessel infiltration, lymphadenopathy and common bile duct deformation, which was primarily considered to be a tumor. Cytological and histological examinations were negative, particularly after the diagnostic laparoscopic revision, multiple biopsies were taken from the pancreatic mass and from the adjacent lymph nodes in the periportal region.

Large metastatic lymph nodes are not typical for pancreatic cancer. Even though the pancreatic head mass was highly suspicious for cancer by radiological examinations, histologically - no signs of malignancy was present, because of the desmoplastic reaction.

Since pathology did not report signs of malignancy, the patient was managed as autoimmune pancreatitis.

Throughout the treatment process the patient's condition was deteriorating, therefore multiple CTs, MRs, and PET/CTs (within a 6-month period 12 cross-sectional examinations of the abdomen) were performed. What was typical for autoimmune pancreatitis at the beginning of the disease:

1) Irregular hypoattenuating mass in the pancreatic parenchyma, which was expanding on the adjacent tissues;
2) Soft-tissue component, which was surrounding the peripancreatic vessels, without vascular lumen distortions;
3) Main pancreatic duct was not dilatated;
4) Pancreatic upstream atrophy was not present;
5) Lymphadenopathy mainly in the porta hepatis;
6) No visceral metastasis.

All upper listed radiological criteria with negative pathology report after diagnostic laparoscopy, was dictating for having autoimmune pancreatitis. As we know from the literature (case reports and articles), type 1 autoimmune pancreatitis is the type that has adjacent peripancreatic tissue infiltration with vessel encasement [19], compared to type 2 autoimmune pancreatitis, which usually exhibits local manifestations of the main pancreatic duct [20]. Another important aspect for type 1 autoimmune pancreatitis is the elevated level of IgG4 in blood, which was within the normal range in our patient.

Since the pathology report was negative for a malignancy, and definite radiological criteria for pancreatic cancer initially were not seen, i.e.

a) Visceral metastasis,
b) Pancreatic parenchymal upstream atrophy,
c) Main pancreatic duct dilatation,
d) Vessel encasement with luminal distortion.

Only radiological criteria that was guiding from the first moment that the patient was admitted, was marked cholestasis, which is typical for both inflammatory and neoplastic conditions.

The desmoplastic reaction is typically seen in pancreatic cancer [2]. Surgical or interventional biopsy specimens sometimes have fibrosis as their pathology report, which has been the reason for delayed cancer diagnosis in our case. In situations, when pathology does not represent the actual condition, radiological work-up with clinical correlation should be graded higher in the decision-making order chain.

Retrospectively in our case, when treatment with steroids was not responding, and the patient's IgG4 level was not elevated, it could have been crucial to change gears, and consider the possibility of malignancy higher than inflammatory. Nonetheless pathology report was graded higher than radiological and the diagnosis of type 1 autoimmune pancreatitis was replaced with possible type 2 autoimmune pancreatitis.

From the literature review we emphasize that type 2 autoimmune pancreatitis is a sole pancreatic condition, and peripancreatic infiltration or vessel encasement is not typical. With that in mind, cancer still becomes more probable, than pancreatitis.

Within 6 months of ineffective treatment of initially type 1 and later type 2 autoimmune pancreatitis, biopsy repetition was recommended, which verified pancreatic adenocarcinoma.

In our case, at the time of diagnosis, all evident radiological criteria present with initial imaging, are typical for both pancreatic cancer and autoimmune pancreatitis, but after 1 month of ineffective treatment (particularly with steroids), the main pancreatic duct started to dilate, and upstream parenchymal atrophy later became apparent. That is a key aspect, and these 2 radiological criteria are typical for pancreatic cancer, and at that point imaging could have changed the understanding of the underlying pathology, and rebiopsy could have been obtained much earlier. By this, we allocate the reader with the prognosticative information and differential diagnosis, thereby providing a tool that should be useful for future decision making in the clinical setting.

## Conclusion

Pancreatic cancer is in the incline of becoming the deadliest cancer, but radiological and pathological diagnostics is still an issue, even though targeted interventional procedures are routinely performed. For the past several years autoimmune pancreatitis has become a separate entity with its subtypes, and are diagnosed quite often in abdominal imaging departments, but unfortunately there are not many scientific papers that will give a strong radiological definition between the autoimmune pancreatitis and pancreatic adenocarcinoma.

In situations when there is a mismatch between histology and radiological follow-up reports, it is the radiologists who should be leading the process of defining the diagnosis. Therefore, diagnostic pancreatology needs more robust criteria for defining pancreatic inflammatory and neoplastic processes.

## Patient consent

Written informed consent was obtained from the patient for publication of this case report.
